# Hepatitis C Virus E2 Protein Ectodomain Is Essential for Assembly of Infectious Virions

**DOI:** 10.4061/2011/968161

**Published:** 2010-10-12

**Authors:** Alessia Bianchi, Stefania Crotta, Michela Brazzoli, Steven K. H. Foung, Marcello Merola

**Affiliations:** ^1^Department of Molecular Immunology, Novartis Vaccines and Diagnostic, Via Fiorentina 1, 53100 Siena, Italy; ^2^Division of Immunoregulation, National Institute of Medical Research, The Ridgeway, Mill Hill, London NW7 1AA, UK; ^3^Department of Pathology, Stanford University, Stanford, CA 94305, USA; ^4^Department of Structural and Functional Biology, University of Naples “Federico II” at MSA, 80132 Naples, Italy

## Abstract

The Hepatitis C virus E1 and E2 envelope proteins are the major players in all events required for virus entry into target cells. In addition, the recently developed HCV cell culture system has indicated that E1E2 heterodimer formation is a prerequisite for viral particle production. In this paper, we explored a new genetic approach to construct intergenotypic 2a/1b chimeras, maintaining the structural region of the infectious strain JFH1 and substituting the soluble portion of E1 and/or E2 proteins. This strategy provides useful information on the role of the surface-exposed domain of the envelope proteins in virus morphogenesis and allows comparative analysis of different HCV genotypes. We found that substituting the E2 protein ectodomain region abolishes the production of chimeric infectious particles. Our data indicate that the soluble part of the E2 protein is involved in a genotype-specific interplay with remaining viral proteins that affect the HCV assembly process.

## 1. Introduction

Hepatitis C virus (HCV) is a positive-strand RNA virus that belongs to the *Flaviviridae *family. Its genome of 9.6 Kb is composed of a 5′ nontranslated region (NTR), a single ORF encoding 10 single products and a 3′ NTR [1]. Individual proteins are generated via co- and posttranslational cleavages mediated by both cellular and viral proteases. The core structural proteins, E1 and E2, reside in the N-terminal region of the polyprotein precursor. They are linked to the replicase proteins NS3–NS5B via p7 and NS2, whose role, even if not completely defined, was recently established as crucial in the assembly/release of nascent HCV particles [2–4]. The poor replication of HCV in cell culture systems has slowed down progress in viral life cycle studies; however, this problem has been partially overcome by the discovery of the infectious properties of the GT2a isolate JFH1 [5].

Relatively efficient production of infectious HCV in cell culture (HCVcc) was initially restricted to genotype 2a, either as the authentic JFH1 isolate or as the intragenotypic Jc1 chimera. Several efforts have been made to extend the HCVcc system by the construction of intergenotypic chimeras. The availability of a spectrum of chimeric HCV genomes differing in their structural region is required for comparative analyses of processes that are governed by such proteins and for the screening of antiviral compounds blocking the early steps of infection that ideally target all HCV genotypes. Intergenotypic chimeras reported so far consist of the 3′-half of the JFH1 genome and the 5′ moiety, including the whole or part of NS2, taken from a different genotype (GTs 1–7) [6]. This strategy was successful in the case of intragenotypic chimera, but it did not allow efficient production of intergenotypic chimeric particles, which suggests that determinants within the structural proteins govern kinetics and efficiency of virus assembly and release. Indeed, as deduced from the reported mapping studies, virus release is most efficient when the JFH1 substituted portion comprises the whole NS2, or part of it, suggesting that genetic interactions between this protein and upstream sequences are required for production of nascent viral particles [7]. 

 Together with the capsid protein, the main components of HCV virion are the envelope proteins E1 and E2, whose roles have been studied using heterologous expression systems and replicon models. Overall, the data obtained indicates that the E1 and E2 glycoproteins interact through their transmembrane domain to form noncovalent heterodimers that represent the putative viral spike [8]. While the transmembrane (TM) anchor region of both E1 and E2 is involved in ER retention and E1E2 association [9], the soluble part plays major roles in the glycoprotein folding process, in virus entry, and in modulating immune responses [10, 11]. In addition, the more recently developed HCVcc system has suggested that E1E2 heterodimer formation is a prerequisite for viral particle production. Indeed, the abolishment of infectious virus production following transfection of the defective JFH1/ΔE1E2 genome has shown that the absence of envelope proteins results in a general block of virion morphogenesis and influences the overall infectivity of secreted particles, as cells transfected with JFH1/ΔE1E2 RNA showed a dramatic reduction of core release [5, 7].

In the present study, we sought to investigate, in the context of an infectious cycle, new potential functions of E1 and E2 envelope proteins in the production of nascent virions. We designed a new strategy to construct two 2a/1b intergenotypic chimeras, in which only the sequences encoding for the E1 and/or E2 glycoprotein ectodomain were swapped from JFH1 to the Con1 strain (genotype 1b). Our rationale was that by leaving the TM domain of the 2a strain, correct interactions with other scaffold proteins required for particle assembly would be maintained. At the same time, the region carrying the HCV epitopes would appear as a component of the 1b strain, allowing a comparative analysis of the impact of the E1 and E2 surface-exposed domains on virus morphogenesis. 

The first chimera we generated contained the ectodomain portion of both E1 and E2 of genotype 1b (JFH1/Con1E1E2). This construct was able to replicate in transfected cells, but it did not allow the production of infectious chimeric particles. The same results were found for the second chimera, JFH1/Con1E2, in which only the E2 ectodomain was swapped from genotype 2a to genotype 1b. This evidence led us to focus our attention on the second chimera in order to better define the role of the envelope protein E2 on nascent virion production. Since in the JFH1/Con1E2 construct E1 and E2 have a different genotypic origin, we first analyzed whether glycoproteins from genotype 2a and 1b could correctly interact allowing heterodimer formation. Next, confocal microscopy analysis, transcomplementation experiments and analysis of infectious particles within transfected cells led us to conclude that the substitution of the E2 ectodomain leads to an abortive particle assembly process. The causes of this genetic incompatibility were explored and are discussed. 

## 2. Material and Methods

### 2.1. Cell Lines

All cell lines were grown in Dulbecco's modified Eagle's medium (DMEM, Gibco) supplemented with 10% fetal bovine serum (FBS, Hyclone), antibiotics, and 2 mM l-glutamine. S6.1 cells, a subclone of Huh 7.5 cells that has cleared the replicon [12] and naïve Huh7 cells were used for HCVcc production and HCV infection, respectively. S6.1/E1E2:2a and S6.1/E1E2:1b packaging cell lines that stably express HCV E1 and E2 glycoproteins genotype 2a and genotype 1b, respectively, were kindly provided by S. Crotta and M. Brazzoli.

### 2.2. Plasmid Construction

The plasmids pUC.JFH1, containing the full-length JFH1 genome (GenBank accession number AB047639); pUC.JFH1ΔE1E2, where the entire E1-E2 coding region has been deleted, and the full-length replicon I389neo/core-30/5.1, encoding for genotype 1b replicon, have been described [13, 14]. The plasmid pUC.JFHCon1E1E2 encodes the full length JFH1 genome with the exception of the E1 and E2 ectodomain regions, both deriving from the analogous portion taken from the GT1b-Con1 strain. To design the Con1E1E2 chimeric construct, a PCR-based strategy was adopted to substitute in the JFH1 sequence the region corresponding to the E1 and E2 protein ectodomain, from aa 192 to aa 329 and from aa 384 to aa 683 for E1 and E2, respectively. Similarly, the plasmid pUC.JFHCon1E2 encodes the full-length JFH1 genome with the region from aa 384 to aa 683 (E2 ectodomain) substituted with the analogous region from the GT1b Con1 strain (See Figure S2 in Supplementary Material available online at doi:10.1155/2010/968161). 

### 2.3. In Vitro Transcription RNA and Electroporation

*In vitro* transcripts of the individual constructs were synthesized as described [15]. For electroporation of HCV RNA into S6.1 cells, single-cell suspensions were prepared by trypsinization of monolayers and subsequent resuspension in complete DMEM. S6.1 cells were washed with phosphate buffered saline (PBS), counted, and resuspended at 6 × 10^6^ cells per ml. Five *μ*g of *in vitro* transcribed RNA was mixed with 400 *μ*l of cell suspension by pipetting and then electroporated with a Gene Pulser system (Bio-Rad, Munich, Germany) in a cuvette with a gap width of 0.4 cm (Biorad) at 975 *μ*F and 270 V. Cells were immediately transferred to 6 ml of complete DMEM, and 150 *μ*l of the cell suspension was seeded per well in a 48-well plate. The cells were trypsinized and passaged every 3-4 days for 8 passages as previously described [16].

### 2.4. Immunofluorescence Staining and Virus Titration

Transfected and infected S6.1 cells were fixed with cold 4% paraformaldehyde for immunofluorescence (IF) analysis and then stained with mouse anti-HCV core antibody 3G1-1 (Chiron corporation, USA), or with the anti-E1E2 polyclonal chimpanzee antiserum Ch-L559 (Chiron Corporation, USA) followed by Alexa Fluor 568-conjiugate goat antimouse (Molecular Probes), or Alexa Fluor 488-conjiugate goat antihuman (Molecular Probes). Replication positive cells were determined by core detection under a fluorescence microscope (Zeiss, Observer.A1). The infectivity titer was determined on Huh 7 naïve cells by end-point dilution and immunofluorescence staining. Typically, 15 *μ*l of supernatant or cell lysate was serially diluted 10-fold in DMEM 10% FCS according to the Spearman and Kaerber fit. Cells were seeded in 96-well plates at a density of 1 × 10^4^ cells per well 24 h prior to inoculation with 100 *μ*l of the diluted supernatant or cell lysate (at least eight wells were used per dilution). Cells were fixed 72 hours postinfection, permeabilized, and core-stained as described above. Virus titer was expressed in particles forming units per ml (pfu/ml) enumerating the positive cells by use of a fluorescent microscope.

### 2.5. Cell Labeling and Pulse-Chase Experiment

Subconfluent 35 mm diameter dishes of S6.1/JFH1 and S6.1/JFHCon1E2 transfected cells (or not transfected as negative control) were starved for 1 hour at 37°C in cysteine- and methionine-free medium. Cells were pulse-labeled in the presence of methionine- and cysteine-free medium supplemented with 350 *μ*Ci ml^−1^ of [^35^S]-labeled methionine and cysteine (ProMix, Amersham Pharmacia Biotech) for 20 min at 37°C. The pulse period was stopped by washing cells twice with PBS. For metabolic labeling, the starving step was skipped and cells were incubated for 6 hours to overnight in the presence of 50 *μ*Ci ml^−1^ of [^35^S]-labeled methionine and cysteine (ProMix). Chase period was initiated by adding complete DMEM supplemented with 2.5 mM unlabeled methionine and cysteine, and incubating cells for different chase times at 37°C. For dithiothreitol (DTT) resistance experiments, the regular chase was followed by 5 min incubation at 37°C in presence of 5 mM DTT. After chase time, cells were placed on ice, then the medium was removed and replaced with ice-cold PBS containing 20 mM N-ethylmalamide (NEM). This alkylating agent blocks free sulphydrilic groups and prevents further oxidation of the cysteines [17]. After 5 min of PBS-NEM incubation, cells were lysed in 900 *μ*l lyses buffer, containing 1% Triton X-100 in HEPES-buffered saline (HBS, pH 7.4) including a protease inhibitors cocktail (Complete, EDTA free, Roche), 1 mM phenylmethylsulfonyl fluoride (PMSF), and 1 mM NEM. After 30 min of incubation at 4°C under agitation, total lysates were centrifuged 20 min at 13,000 × g to pellet nuclei and cellular debris. Postnuclear supernatants (PNSs) were used for immunoprecipitation with specific anti-E2 antibodies.

### 2.6. Antibodies, Immunoprecipitation and SDS-PAGE

The two specific anti-HCV E2 antibodies used in this study are the human monoclonal conformational anti-E2 CBH-2 and CBH-5. They are two of a panel of nine conformation sensitive antibodies generated from peripheral B-cells of an asymptomatic HCV-infected individual [18]. These antibodies do not recognize denatured E2, inhibit binding of E2 to the putative receptor CD81, and specifically recognize properly folded E2 (GT2a and 1b, resp., for CBH-2 and CBH-5). PNSs were precleared on protein G coupled to Sepharose Fast Flow (Amersham Pharmacia Biotech) for 1 h at 4°C and were subsequently immunoprecipitated with 1 *μ*g of CBH-2 or CBH-5 at 4°C overnight in presence of 10 *μ*l Dynabeads Protein G (Dynal Biotech ASA). The radioactive counts of the TCA-precipitated fraction were determined on 2 *μ*l of each precleared lysate. Equivalent amounts of total labeled proteins were used for CBH-2 or CBH-5 anti-E2 immunoprecipitations. The immunocomplexes were collected with the proper magnetic device, washed three times with lysis buffer, and solubilized at 95°C for 5 min in 20 *μ*l of SDS loading buffer and separated on SDS-PAGE, followed by autoradiography. For SDS-PAGE in reducing conditions, DTT 100 mM final was added to the samples.

### 2.7. Endoglycosidase H Treatment

Endoglycosidase H (EndoH) cleaves ER type N-linked high mannose sugar chains between the two N-acetylglucosamine residues, thus eliminating most of the contribution of the glycan to the apparent molecular weight in SDS electrophoresis [19]. Immunoprecipitated proteins recovered from beads were incubated with 50 mU endoH in 250 mM sodium acetate pH 5.5, in a final volume of 0.25 ml. The reaction was performed for 6 hours at 37°C in presence of 1 mM PMSF. Digested proteins were added to loading buffer with (reducing) or without (nonreducing) 100 mM DTT for SDS-PAGE analysis.

### 2.8. Confocal Analysis

S6.1/JFH1 and S6.1/JFHCon1E2 cells (or S6.1 not transfected cells as negative control) were plated on 30 mm coverslips in 24-well plates at a density of 5 × 10^4^ cells per well. One day after seeding, cells were fixed in 4% formaldehyde, permeabilized with 0.1% Triton X-100 in PBS, pretreated with blocking solution (0.5% bovine serum albumin in PBS) and incubated at room temperature with the primary antibodies diluted in blocking solution. After three washes in PBS, AlexaFluor-conjugated secondary antibodies were added to the cells. Lipid droplets were stained in paraformaldehyde-fixed cells by briefly rinsing coverslips in 60% propan-2-ol followed by incubation with 0.5 ml 60% propan-2-ol containing oil red O (final concentration approximately 0.6%) for 1.5 to 2 min at room temperature. Coverslips were briefly rinsed with 60% propan-2-ol and washed with PBS and H_2_O. The oil red O staining solution was prepared from a saturated stock of approximately 1% oil red O (Sigma) dissolved in propan-2-ol. Before staining, the stock was diluted with H_2_O and then filtered [20]. Coverslips were mounted in aqueous mounting medium Vectashield. Confocal microscopy was performed with a Bio-Rad 2100 Confocal Microscope using 60x oil or 100x oil objectives. Image analysis was performed using the standard operating software provided with the microscope.

### 2.9. Cell Lysate Preparation, Sedimentation Equilibrium Gradient, and Western Blot Analysis

S6.1/JFH1 and S6.1/JFHCon1E2 cells were washed once with PBS and incubated with trypsin-EDTA for 2 min at 37°C. Cells were resuspended in PBS and collected by centrifugation at 1,500 rpm for 3 min. The cell pellet was resuspended in DMEM-10% FCS, and cells were lysed by four freeze-thaw cycles in liquid nitrogen and a 37°C water bath, respectively. Cell debris was pelleted by centrifugation for 5 min at 4,000 rpm. The supernatant was collected and overlaid onto a discontinuous sucrose gradient. Gradients were formed by equal-volume (750 *μ*l) steps of 20%, 30%, 40%, 50%, and 60% sucrose solutions in TNE buffer (10 mM Tris-HCl, pH 8, 150 mM NaCl, 2 mM EDTA). 250 *μ*l of each supernatant were overlaid on the gradients, and an equilibrium was reached by ultracentrifugation for 16 hours at 36,000 rpm (135,000 × g) in an SW60Ti rotor at 4°C in a Beckman L8-80 M preparative ultracentrifuge. After ultracentrifugation, gradient fractions were collected from the top and titrated for virus infectivity as previously described. The density of the fractions was determined by measuring the refractive index of 10-*μ*l aliquots of each sample. 

For immunoblot analysis, samples were added to SDS-loading buffer, heated for 5 min at 95°C, and loaded onto a 10% polyacrylamide-SDS gel. Primary antibodies used were core-specific mouse monoclonal antibody (3G1-1) at a 1 : 500 concentration, polyclonal antiserum Ch-L559 against E1 and E2 proteins at a 1 : 1000 dilution, and the mouse monoclonal MMM33 anti-NS3 (Novocastra Lab. Ltd. anti-NS3 antibody) at a 1 : 200 dilution. Bound antibodies were detected after a washing step with the ECL plus Western blotting detection system (GE Healthcare).

## 3. Results

### 3.1. Construction and Characterization of Intergenotypic 2a/1b HCV Genomes

**Figure 1:**
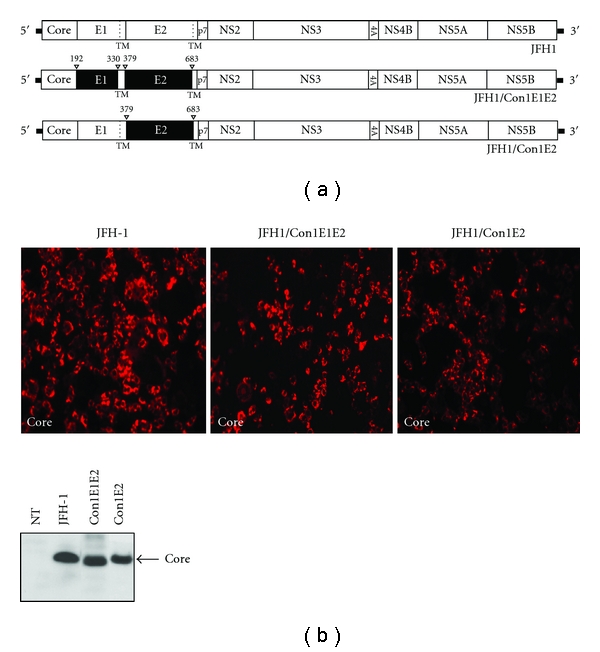
Chimeric JFH1/Con1E1E2 and JFH1/Con1E2 constructs are replication-competent. Schematic representation of the constructs used in this study (a). JFH1-derived 5′ and 3′ nontranslated regions are drawn as thick black lines and JFH1 proteins are depicted as open boxes. JFH1/Con1E1E2 and JFH1/Con1E2 comprise chimeric HCV E1 and E2 proteins consisting of Con1 (black boxes) fused with JFH1 at the indicated positions. (b) Replication of JFH1 and given mutants. S6.1 cells were fixed 72 hrs posttransfection, permeabilized, and incubated with mouse anticore mAb (3G1-1). Cells were then stained with antimouse-AF-568 secondary antibody (red) and replication-positive cells were visualized by core-specific immunofluorescence. Western blot analysis of PNSs from JFH1, Con1/E1E2, and JFH1/Con1E2 transfected cells and from not transfected cells (NT) is also reported on the bottom. The band of ~20 Kda resulted from SDS-PAGE correspond to core protein.

E1 and E2 envelope proteins associate to form noncovalent heterodimers that have been indicated as the major players for virus entry into target cells [10]. This dimerization is also involved in processes that lead to the production of nascent virions, since it has been shown that deletion of the E1 and E2 coding region blocks infectious particle morphogenesis [5]. In this study, we sought to further investigate the production of nascent virions by using a new strategy to design two 2a/1b intergenotypic chimeras. To verify whether E1 and E2 proteins are involved in the assembly process, a system in which modifications of one or both envelope proteins could be introduced without affecting viral RNA replication or heterodimer association was required. To achieve this goal, we replaced the region corresponding to the E1 and/or E2 ectodomain in the assembly-competent JFH1 genome with the same portions taken from the GT1b isolate Con1 (Figure 1(a)). We speculated that since heterodimerization determinants have been ascribed to the TM regions of both glycoproteins, the swapping of the “soluble” domains should not affect the formation of the complex. Furthermore, leaving the TM domain of the 2a strain intact, we allowed interactions to be maintained between the envelope proteins and other proteins of the scaffold that might be required for particle assembly. On the other hand, by generating a viral genome carrying the surface-exposed portion of E1 and/or E2 derived from a heterologous strain (GT 1b), our system allowed a comparative analysis of the impact of the ectodomain moieties on virion morphogenesis. 

For E1/E2 swapping, we targeted the soluble domains excluding the “membranotropic” regions. To achieve this, we submitted the C-terminus regions of both glycoproteins to secondary structure prediction analysis based on two algorithmics, j-pred and phy-pred. We found that for both proteins, the region with hydrophobic characteristic is wider than that reported in the literature. In particular, in the predicted structures the membrane spanning region starts at position 330 instead of 353 for E1 and initiates at residue 683 instead of 718 for E2 (see Figure S1 in Supplementary Material). Although the validation of this analysis was beyond the scope of this work, we based our PCR strategy to substitute the E1 and/or E2 ectodomain on this novel configuration, thus obtaining the JFH1/Con1E1E2 and JFH1/Con1E2 constructs, as described in Figure S2 in Supplementary Material.

To characterize the engineered HCV-JFH1 genomes, S6.1 cells were transfected with JFH1/Con1E1E2 and JFH1/Con1E2 chimeric RNAs in parallel with the wild type genome, which was used as a control in all experiments in this study. Cell culture media were harvested from 72 hours after transfection, and each cell type was checked for the presence of the viral core by immunofluorescence staining. In parallel, cell lysates were submitted to immunoblot analysis. The positive cells showed core-specific cytoplasmic staining patterns as previously described [21]. In all samples, replication appeared to be efficient in about 80%–90% of cells, indicating that the substitution of the ectodomain region does not affect HCV replication (Figure 1(b)). The immunoblot obtained on cell lysates using the anticore antibody revealed a single band of approximatively 20 KDa consistent with the molecular weight of the protein (Figure 1(b), bottom panel). The core signal is comparable in all samples, further confirming that there are no differences in terms of replication ability between wild-type and chimeric constructs. Next, in order to verify the release of infectious particles by S6.1 transfected cells, cell supernatants were collected at different time points, from 3 to 30 days posttransfection, and tested for infectivity by inoculation onto Huh7 naïve cells. After 72 hours, infected cells were fixed, permeabilized, and examined for foci of cells expressing HCV core protein by using indirect immunofluorescence. A proper titration was possible solely for the S6.1/JFH1 cells since none of the chimeric constructs allowed the production of infectious particles (data not show). 

As the ability of the viral RNA to replicate was not affected by the glycoprotein exchange, the ectodomain swapping most likely affects one or more of the steps downstream RNA replication. Based on this consideration, we performed a series of experiments to evaluate which process is influenced by the substitution of the E1 and E2 ectodomain-coding region in the JFH1 scaffold. It is reported that the E1-core protein interaction is important for a proper viral particle assembly process, as E1 associates with the core protein through a cytoplasmic loop containing five amino acids (312–315) [22]. To exclude the possibility that the observed lack of infectious particle production by our chimera was simply due to the inability of Con1-E1 to interact with the capsid protein, we decided to focus our attention on the JFH/Con1E2 chimera in which the original JFH1-E1 sequence is maintained. Regarding the E2 protein, it is worth noting that its transmembrane region is reported to play a key role in the coordinated assembly and reorganization of the E1E2 heterodimer [23]. However, in our chimeric JFH1/Con1E2 construct, the E2 TM region is derived from the genotype 2a, as well as the E1 protein. In this way, the ability of the chimeric E2 to dimerize with E1 JFH1-derived protein should be maintained.

### 3.2. Folding and Maturation Process of Chimeric E1-2a/E2-1b Heterodimers

**Figure 2:**
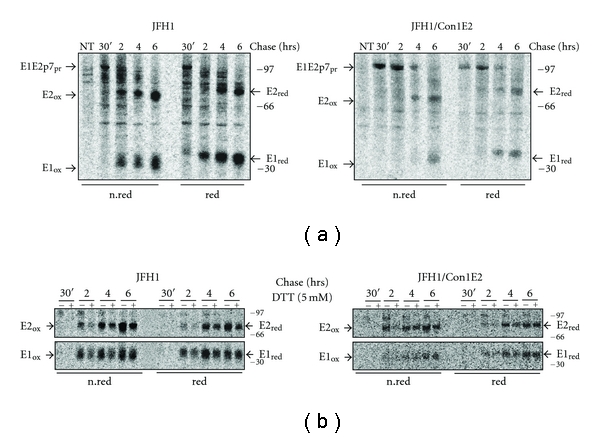
Oxidation kinetics and DTT-resistance analysis of HCV proteins E1 and E2. (a) S6.1 cells transfected with JFH1 or JFH1/Con1E2 RNA were pulse labeled with [^35^S] methionine and cysteine for 20 min and chased for different time periods, from 30 min to 6 hrs. PNSs were immunoprecipitated with a conformational monoclonal anti-E2 antibody (CBH-2 and CBH-5 for genotypes 2a and 1b, resp.) and analyzed on SDS-PAGE. Chase periods are reported above the lanes. Samples were analyzed on 10% SDS-PAGE under nonreducing (n.red) and reducing (red) conditions (DTT 200 mM). (b) S6.1 cells transfected with JFH1 or JFH1/Con1E2 RNA were pulse labeled and chased for the indicated time periods, in duplicate. One of the dishes in each pair was chased for an additional 5 min in the presence of 5 mM DTT (+) in the culture media before proceeding with lysis. PNSs were immunoprecipitated with CBH2 or CBH5 for genotype 2a and 1b E2 proteins, respectively. Upper panel, E2 protein. Lower panel, E1 protein. The samples were analyzed on 10% SDS PAGE under reducing and nonreducing conditions. Symbols refer to different form of E1 and E2: ox for oxidized; red for reduced; pr for protein precursor; NT for not transfected. Position on gel of prestained Molecular Weight marker (Amersham) is reported on the right side.

The E1 and E2 proteins are targeted to the ER by a signal sequence and are cotranslationally separated from each other by host signal peptidase cleavage. In the ER, they acquire several N-linked oligosaccharide chains and assemble into noncovalently bound E1E2 heterodimers that represent the functional units of the HCV spike [24]. To analyze the formation of the heterodimer in our system, we tested several anti-E2 conformational mAbs in immunoprecipitation of metabolic labeled S6.1/JFH1 wild type (wt) and S6.1/JFHCon1E2 cell lysates, and checked for the presence of coimmunoprecipitated E1. We found that E2 was immunoprecipitated by the conformational monoclonal antibodies CBH-2 and CBH-5 for the JFH1- or Con1-derived proteins, respectively (Figure 2(a)). The simple observation that CBH-5 recognized E2 from genotype 1b and coimmunoprecipitates E1 from genotype 2a attested that glycoproteins from different genotypes are able to form heterodimers. 

To better define the behaviour of newly synthesized E1 and E2, S6.1/JFH1 and S6.1/JFHCon1E2 cells were pulse-labeled for 20 min and chased for different time periods. Figure 2(a) shows the oxidative status of immunoprecipitated E2 and coimmunoprecipitated E1 from 30 min to 6 hours after synthesis. Concerning the wild type species (Figure 2(a), left panel), the formation and recognition of the conformational epitope on E2 is weakly revealed 30 min after synthesis, becoming more evident after 2 hours. Immunoprecipitation of E2 with the conformational Ab always carries down E1, indicating that the formation of the conformational epitope on E2 is contemporary, or follows, the formation of the E1E2 heterodimers. We observed a similar pattern for both the wt and the chimeric E1E2 heterodimers (Figure 2(a), right panel), indicating that the Con1-derived E2 portion interacts with the E1 glycoprotein encoded by JFH1. In both gels, a slower migrating band of about 100 Kda is also seen, which most likely represents the unprocessed E1E2 precursor, still associated to p7 as described in other reports [25, 26]. The half life of this species, that inversely correlate with the appearance of mature E2, is the most remarkable difference between the wild type and the chimeric samples. In fact, by comparing the two panels of Figure 2(a), it is evident that after 4 hrs of chase the chimeric precursor is still present while the wild type one has completely disappeared. The different kinetics of the E1E2p7 precursor processing and the parallel slower appearance of the chimeric E1E2 heterodimer suggests a more complex process of association between E1 and E2 proteins that are derived from different genotypes. 

To further assess the oxidation status of E2 in the different steps of wt and chimeric heterodimer folding kinetics, we performed a DTT-sensitivity assay on both samples. In completely folded proteins, disulfide bonds are protected from reduction by exogenously added reducing agents such as DTT. Conversely, in incompletely folded species, the S-S bonds are accessible and the treatment will restore the reduced state of the cysteines [25, 27]. To perform this experiment each chase-time was done in duplicate, with one of the two samples treated with DTT for 5 min before the end of the chase-period. Normalized samples were immunoprecipitated and HCV glycoproteins separated by SDS-PAGE (Figure 2(b)). Since we used conformational anti-E2 antibodies that recognize DTT-sensitive epitopes, the un-oxidized form of E2 will not be immunoprecipitated in presence of the reducing agent and, therefore, will not be present on the gel. The relative amount of DTT-resistant native species can, therefore, be evaluated by comparing the intensity of the E2 bands in a DTT treated sample versus the untreated sample. Figure 2(b) shows that the behaviour of E2 is similar when derived from JFH1 or Con1, showing a time-dependent increase of DTT-resistance that is almost complete at the 6 hour chase-point. Since the achievement of a DTT resistant conformation is a strong indication that the protein is in its native state, this data supports the previous conclusion that GT1b-E2 is able to complete its oxidative folding also in a different genotypic context, such as the JFH1 genome. 

**Figure 3:**
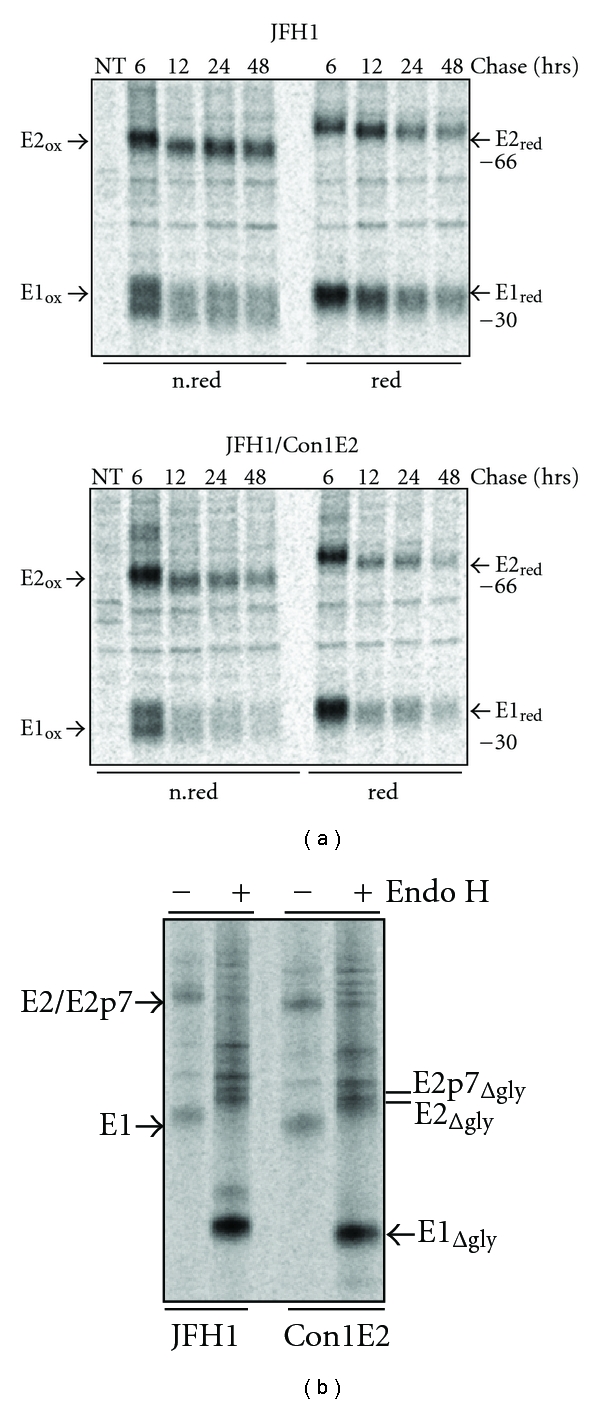
Oxidation kinetics and glycosylation processing of E1E2 heterodimer. (a) S6.1 cells transfected with JFH1 or JFH1/Con1E2 RNA were pulse labeled with [^35^S] methionine and cysteine for 20 min and chased for different time periods, from 6 hrs to 48 hrs. PNSs were immunoprecipitated with a conformational monoclonal anti-E2 antibody (CBH2 or CBH5, resp., for genotype 2a and 1b) and analyzed on SDS-PAGE. Chase periods are reported above the lanes. Samples were analyzed on 10% SDS-PAGE under nonreducing and reducing conditions (DTT 200 mM). (b) Transfected cells were metabolically labeled for 6 hrs in presence of [^35^S] Met and Cys. E1 and E2 proteins were immunoprecipitated from the PNS with CBH-2 or CBH-5, respectively, for wt and chimeric species, treated (+) or not (−) with endo H and analyzed on 10% SDS-PAGE under nonreducing conditions. Note that Con1-derived proteins display an higher electrophoretic mobility than E1E2 proteins from JFH1 transfected cells. Symbol Δgly is for deglycosylated proteins.

However, the slower maturation of the chimeric protein precursor led us to speculate that it could accumulate in the ER-compartment where, if not correctly processed, it could be targeted to the proteasomal pathway. We addressed this question by extending the chase period to 48 hours for both S6.1/JFH1 and S6.1/JFHCon1E2 cells (Figure 3(a)). Although the Con1-derived E2 was cleared to a higher extent than the wt, the difference was not dramatic and the absence of smeared E1 and E2 bands was consistent with a successful folding process. In addition, inhibitors of the proteasomal degradation pathway such as Lactacystein and MG132 did not influence the amount of E1 and E2 recovered at the later chase-time (data not show). This provides further evidence against a proteasomal degradation mechanism and excludes the possibility that E1 and E2 glycoproteins are degraded due to slower heterodimerization and, therefore, not available for incorporation into nascent chimeric particles. 

Comparative analysis of the glycosylation status of both wild type and chimeric heterodimers also ruled out differences in posttranslational modification between the two samples (Figure 3(b)). A peculiar characteristic of HCV envelope glycoproteins is the high extent of N-linked glycans at positions strongly conserved along the different genotypes, and the absence of post-ER types of modification [8]. Metabolically labeled S6.1/JFH1 and S6.1/Con1E2 cell lysates were immunoprecipitated, and the proteins recovered from the beads were incubated in the presence or absence of the Endo H enzyme, which is a common treatment to analyze the glycosylation status of proteins [19]. The digested proteins were then analyzed by SDS-PAGE in reducing conditions. As shown in Figure 3(b), in both samples the E1 and E2 proteins migrate considerably faster after Endo H digestion, consistent with a complete deglycosylation. Upon Endo H treatment, we could visualize a splitting of the band corresponding to E2 protein. This is consistent with the simultaneous presence of two species, the mature E2 and the E2p7 precursor, which has already been described in previous reports [25, 26]. Analysis of the glycans bound to HCV envelope glycoprotein have indicated that only high-mannose type oligosaccharides are associated with these proteins, thus excluding that, as for the wild type, the chimeric heterodimers have transited through the Golgi compartment [28].

### 3.3. Subcellular Localization of Viral Structural and Nonstructural Proteins in S6.1/JFH1 and S6.1/Con1E2 Transfected Cells

Folding analysis and deglycosylation studies support the idea that the chimeric E1E2 undergoes correct maturation and association processes leading to the formation of functional heterodimers, crucial for the infection ability of the nascent viral particles. As a consequence, our next attempt was to clarify the subsequent step in the viral particle generation, namely, the assembly process. To date, there is little understanding of the mechanism underlying the assembly process of HCV in cultured cells. However, recent studies have assessed the role of the cellular lipid droplets (LDs) as potential assembly sites. In particular, taking advantage of the JFH1 culture model, it was demonstrated that the HCV core and NS5a perfectly colocalize with LDs, showing a ring-like pattern that corresponds to the surface of LDs [29].

**Figure 4:**
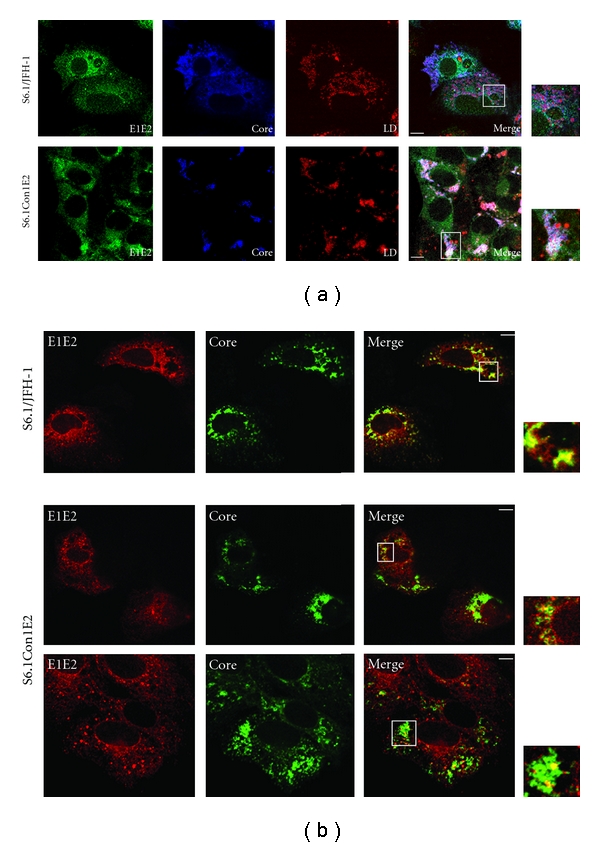
Intracellular distribution of HCV structural proteins. Transfected S6.1/JFH1 and S6.1/Con1E2 grown on coverslips were fixed and stained with the anti-E1/E2 chimpanzee antisera L559 (green) and the mouse monoclonal antibody 3GI-I (blue). Lipid droplets were stained with oil red O. The merge images and the corresponding magnification are shown on the right. (a) Intracellular distribution of E1/E2, core and lipid droplets. (b) Relative intracellular colocalization of E1/E2 and core proteins. For S6.1/Con1E2 cells, two different populations are showed in panel (b). Bars, 20 *μ*m.

In this study, we investigate the subcellular localization of HCV E1 and E2 envelope proteins in order to evaluate potential differences in the viral protein distribution between wild type and chimeric transfected cells that might be the cause of a deficient assembly process. Transfected cells were grown on glass coverslips, fixed with paraformaldehyde and processed for indirect immunofluorescence with specific antibodies against E1E2, core and NS5a proteins, and stained with oil red O to specifically mark lipid droplets. As shown in Figure 4(a), E1E2 heterodimers are distributed in a comparable way in both JFH1 and JHF1/Con1E2 transfected cells. In particular, they show a pattern of specific fluorescence in a network of cytoplasmic membranes and at the nuclear periphery. This is consistent with previous data obtained by using the HCVcc system [21] and definitively confirms that E1/E2 proteins are retained in the ER compartment, as already shown in other studies performed with diverse heterologous expression system [30, 31]. Concerning the capsid protein, the staining in both S6.1/JFH1 and S6.1/Con1E2 cells reveals an organization in ring-like structure, already reported in the context of cells transfected with the infectious JFH1 genome [29, 32]. When lipid droplets are labeled with oil red O the association between the core protein and LDs is clear (see merge panels Figure 4(a)). Furthermore, in both wt and chimeric species most of the core localizes on the LDs surface (see zoomed panel Figure 4(a)), but the cellular distribution of such complexes differs in the two cell types. More precisely, in S6.1 transfected with the JFH/Con1E2 genome, associated LDs-core mostly accumulate in the apical periphery of the cell. By contrast, in almost all cells transfected with the wt genome, LDs-core coated do not cluster but rather are distributed throughout the cytoplasm. To gain more accurate information about the relative localization of structural proteins, we explored the colocalization of the E1E2 heterodimer with core and revealed an additional slight difference between the two cell types. In fact, while not evident in S6.1/JFH1 cells, in a minor subset of S6.1/Con1E2 cells (roughly 20%), we detected a different pattern of E1E2 distribution locally concentrated in dot-like structures (Figure 4(b), bottom panel). In these structures, E1E2 co-localize with the core protein (see merge on the bottom panel). It is worth noting that such structures, in which all the viral components accumulate, are typical of the replicon system, that was often used in HCV research, but that cannot support the production of infectious particles [33]. We speculate that these pointed structures could represent sites of storage of viral proteins that cannot be incorporated into nascent virions, thus hindering release of the virus. Indeed, a rapid assembly process should result in a rapid liberation of HCV proteins from LDs and would not result in an accumulation of structural proteins in the periphery of the cell, as observed in S6.1/Con1E2 cells [32].

**Figure 5:**
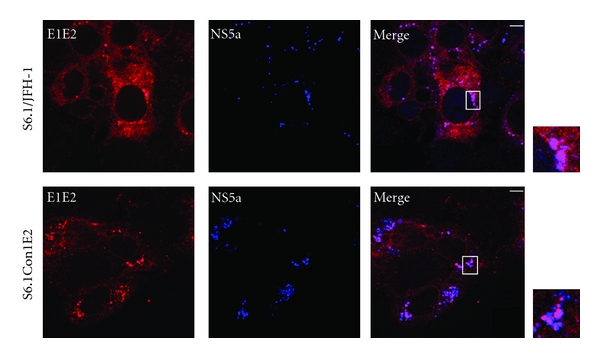
Intracellular distribution of HCV structural and nonstructural proteins. Transfected S6.1/JFH1 and S6.1/Con1E2 grown on coverslips were fixed and double-stained with the anti-E1/E2 chimpanzee antisera L559 (red) and the anti-NS5 rabbit monoclonal antibody (blue). The merge images and the corresponding magnification are shown on the right. Bars, 20 *μ*m.

In addition to the core, it has recently been proposed that NS5a plays a significant role in the assembly of HCV particles. In particular, it has been proposed that NS5a is involved in the recruitment of the replication complex on the LDs surface, an indispensable event to trigger particle formation [34, 35]. As shown in Figure 5, in S6.1/JFH1 cells, E1 and E2 and the nonstructural protein NS5a co-localize in the membranous web, which is the hypothetical site of the replication complex, confirming that the replication and the assembly factories might be located within the same region. In S6.1/Con1E2 cells, NS5a localization appeared more spot-like, with the optimal co-localization with E1E2 heterodimer corresponding to the dot-like structures described above.

### 3.4. S6.1/Con1E2 Cells Do Not Contain Immature Infectious Viral Particle

Confocal microscopy analyses suggest that there is an inverse correlation between the efficiency of virus production and LDs-core protein clustering within the cells. To further investigate if these observations reflect a real functional difference in terms of particle production, we verified whether chimeric particles are correctly assembled and then accumulated in S6.1/Con1E2 cells. Recent studies reported the existence of infectious HCV particles within hepatoma cells transfected with the JFH1 genome. In such studies, it was demonstrated that the intracellular particles, considered as immature viruses, exhibit a different buoyant density with respect to the particles secreted in the milieu, but that they are already infectious when used to inoculate Huh7 naïve cells [36, 37]. If intracellular immature virions are formed by our chimeric proteins, then the defect could be ascribed to the release step. On the contrary, in the case of infectious particles which are not found within S6.1/Con1E2 cells, the conclusion should be that the assembly process itself cannot proceed to maturation.

**Figure 6:**
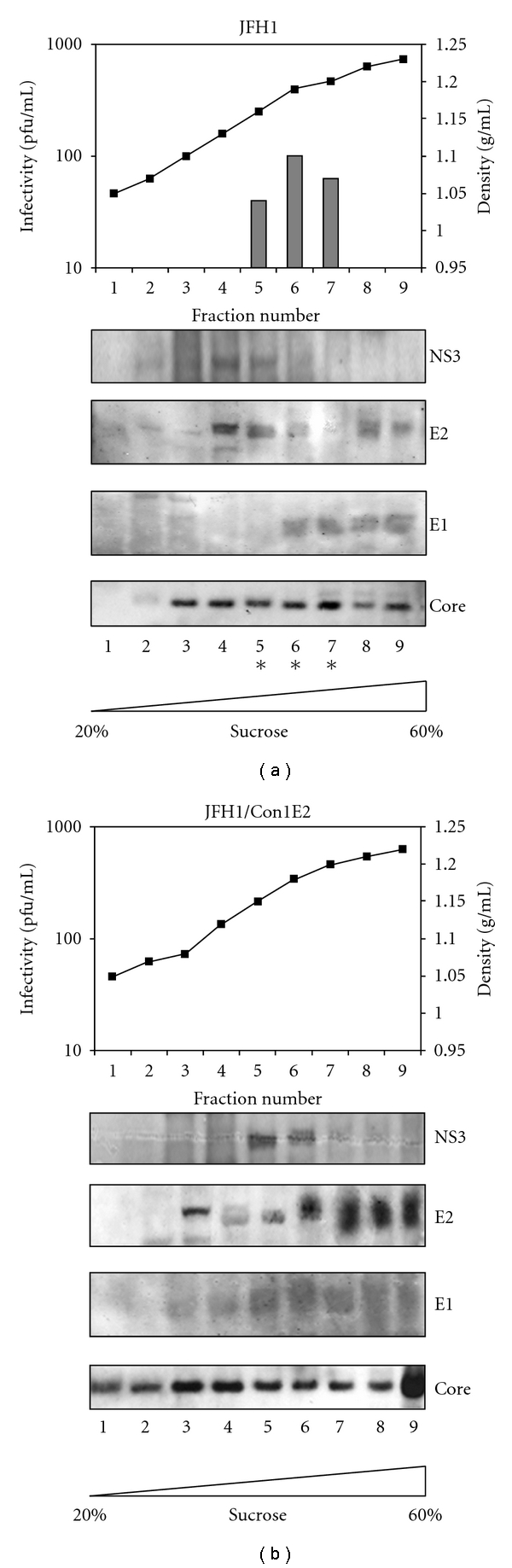
Sedimentation equilibrium analysis. Buoyant density profile of S6.1/JFH1 (a) and S6.1/Con1E2 (b) cell lysates determined by equilibrium ultracentrifugation in sucrose gradient is reported on the top panel. Fractions of the 20%–60% gradient were collected from the top, and infectivity was determined by serial dilution and immunofluorescence staining anticore protein. The infectivity (pfu/ml) is shown as a bar chart. The density (g/ml) of each fraction is shown as a dotted line. Each sucrose fraction was processed by Western blot analysis as reported below the corresponding chart. The mouse monoclonal MMM33 anti-NS3 was used to detect the HCV nonstructural protein NS3, E1 and E2 proteins were revealed by the polyclonal Ch-L559 antisera and core protein was detected by the mouse monoclonal antibody 3G1-1.

To release intracellular infectious particles, S6.1/JFH1 and S6.1/Con1E2 cells were lysed by four freeze-thaw cycles and the PNS were loaded onto a discontinuous sucrose gradient (20% to 60% in TNE buffer). As reported in Figure 6(a), in S6.1 cells transfected with the wild type genome, we were able to detect cell-associated infectivity in fractions corresponding to densities ranging from 1.15 to 1.21 g/ml. This data is in agreement with that reported in the literature and it is consistent with the presence of intracellular infectious particles. By contrast, none of the fractions collected from S6.1/Con1E2 cell lysates showed any infectivity (Figure 6(b)). The lack of infectious particles inside the cells transfected with the chimeric genome confirms the idea that the E2 ectodomain exchange affects the assembly process rather than the following release step. The Western blot analysis of the density-separated species was pursued to check the presence of the structural proteins (Core, E1, and E2) and the nonstructural NS3 in each fraction, for both S6.1/JFH1 and S6.1/Con1E2 transfected cells. Comparing the pattern of distribution of the viral structural proteins, we noted an accumulation of core and E2 proteins at the bottom of the gradient (fraction 9) in the case of S6.1/Con1E2 cell lysate (Figure 6(b)) that was not observed in the wt sample (Figure 6(a)). This evidence indicates that a consistent portion of the capsid, as well as the E2 protein, is present as high molecular weight complexes that sediment at high concentrations of sucrose. These complexes likely represent nonfunctional aggregates, probably composed mainly of proteins (core and other viral and cellular proteins) and of cellular membranous structures. Finally, the more diffuse shape of the E1 and E2 bands in the chimeric sample is consistent with the presence of heterogeneous species that are most likely incorrectly folded forms of the two proteins (Figure 6(b)). This observation strongly indicates that the E2 ectodomain swapping generates a remarkable amount of misfolded glycoproteins whose permanence inside the cell could in part explain the impairment of particle assembly. It is worth noting that these dead-end species were not revealed in immunoprecipitation (IP) using conformational anti-E2 antibodies but were recognized by an anti-E1/E2 antisera. Indeed, differently from the monoclonal CBH-2 and CBH-5 antibodies, the polyclonal Ch-L559 antisera recognized all forms of E1 and E2 in Western blot analysis [38, 39].

### 3.5. The JFH1/Con1E2 Defective Genome Can Be Rescued by the transcomplementation of the GT2a-E2 Ectodomain Region

**Figure 7:**
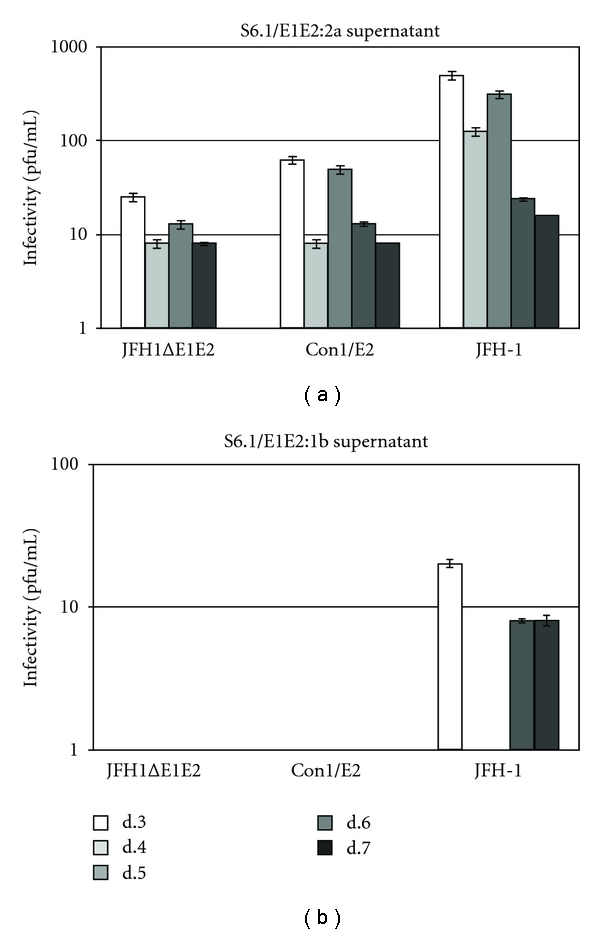
Infectivity of transcomplemented JFH1ΔE1E2 and JFH1/Con1E2 defective viral genomes. *In vitro* transcribed genomic JFH1ΔE1E2, Con1E2 defective RNAs and the full-length JFH1 genome were delivered to S6.1/E1E2:2a (a) and S6.1/E1E2:1b (b) packaging cell lines that stably express E1 and E2 HCV proteins of genotype 2a and 1b, respectively. From day 3 to day 7 after transfection, cell supernatants were collected and tested for infectivity (pfu/ml) by serial dilution and immunofluorescence against core protein.

Overall, the data shown so far attest that the interchange of E2 ectodomain profoundly alters the efficiency of viral assembly at early stages of particles formation. These observations led us to suppose a genetic incompatibility between the genotype 1b E2 protein and genotype 2a structural proteins. We further investigated this possibility by designing an experiment that takes advantage of the “flexibility” of the HCV RNA genome. In fact, the assembly of HCV progeny viruses can be achieved providing a nuclear constitutive expression of the entire structural region, from core to NS2 protein, to a subgenomic replicon [40, 41]. Therefore, considering our chimeric genome as defective for some assembly functions, we sought to rescue the production of infectious particles by providing the lacking functions *in trans*. To carry out this experiment, we used two different S6.1 packaging cell lines that stably express the HCV E1 and E2 envelope proteins, one from genotype 2a and the other from genotype 1b, designed S6.1/E1E2:2a and S6.1/E1E2:1b, respectively. We transfected each cell line with the wt and chimeric JFH1/Con1E2 RNAs, and we used the JFH1ΔE1E2 construct as a control for the transcomplementation. Supernatants from transfected S6.1/E1E2:2a and S6.1/E1E2:1b cells were collected from day 3 to day 7 and each one was titrated by inoculating Huh7 naïve cells. As expected, the JFH1 full-length genome allows the production of infectious particles, confirming that both packaging cell lines support HCV replication, assembly, and release (Figure 7). However, when comparing the virus titers obtained from S6.1/E1E2:2a (Figure 7(a)) and S6.1/E1E2:1b cells (Figure 7(b)) transfected with the wt RNA, a substantially difference in terms of productivity clearly appears. In fact, the HCVcc titer reached in the S6.1/E1E2:2a supernatant was approximately 10 fold higher than that obtained from S6.1/E1E2:1b (Figure 7(a) versus Figure 7(b)). If the hypothesis of genotypic incompatibility is true, we might expect that E1E2 from genotype 1b could compete with E1E2 from genotype 2a in interacting with the other structural proteins from genotype 2a, thus leading to an abortive assembly process. The lower viral titer with respect to the one reached from S6.1/E1E2:2a cells would thus be explained as displacement of genotype 2a E1E2 by the glycoproteins expressed by S6.1/E1E2:1b. As evident in Figure 7, the chimeric genome JFH1/Con1E2 shows the same behavior as the defective construct JFH1ΔE1E2, confirming that the swapping of the E2 ectodomain is equivalent to the deletion of the E2 envelope protein coding region. Importantly, only in S6.1/E1E2:2a cell supernatants, we recovered infectious particles upon transfection with both JFH1ΔE1E2 and JFH1/Con1E2 RNAs (Figure 7(a)). This clearly indicates that the transcomplementation is possible, but only when the packaging cell provides autologous envelope proteins. Indeed, as evident by the absence of infectious particles in the supernatant, S6.1/E1E2:1b cells are not able to rescue the defective assembly function of neither JFH1ΔE1E2, nor of the JFH1/Con1E2 genome (Figure 7(b)).

## 4. Discussion and Conclusion

The recent development of the full HCV infectious system (HCVcc) by Wakita and colleagues for the JFH1 strain [5] has represented a big breakthrough in HCV research since it allows to study the complete life cycle of HCV and to define the roles of proteins that are not required for viral RNA replication. Following this achievement, several efforts have been made to extend this cell culture system to all HCV genotypes, but these attempts have ended with the conclusion that for unknown reasons, the JFH1 backbone is absolutely required. To overcome the restriction to JFH1 strain, chimeric viruses of representative HCV strains belonging to genotypes 1 to 7 [6] have been generated. Commonly reported chimeras consist of the 3′-half of the JFH1 genome and the 5′-moiety of the other strain, extending to p7 and part or whole of NS2. However, while the construction of intragenotypic chimera was highly successful, several reports pointed out that the virus yield obtained using intergenotypic chimeras was reduced and often required adaptive mutations for the establishment of a cell culture system. 

In the present study, we explored the possibility of establishing a general procedure to obtain cell culture chimeric infectious particles by strain-swapping the soluble domain of the E1 and E2 envelope proteins. This strategy would allow genotypic-specific studies of therapeutics that target viral entry steps, such as neutralizing monoclonal antibodies or fusion inhibitors and would provide useful information on viral assembly determinants. The rationale underlying our approach was the following: E1 and E2 soluble moieties are the only domains that face into the ER-lumen and are thus exposed on the surface of mature virions. The remaining HCV proteins are either anchored to the cytoplasmic side of the ER membrane or, in the case of core, are localized on the surface of lipid droplets. The C-terminus region of NS2 is still a matter of debate since its topology has not yet been determined. According to a recent model of virus assembly proposed by Miyanary et al. [32], all interactions necessary for the formation of the nascent particles should involve the cytoplasmic oriented domains or the TM regions. Since in our constructs all these regions were conserved from the JFH1 strain, the swapping of the envelope protein ectodomain should not interfere with the assembly mechanism. From these considerations, we have constructed and analyzed two novel intergenotypic HCV chimeras, still based on the JFH1 strain backbone, with only the ectodomain of the E1 and/or E2 envelope proteins substituted with the analogous region taken from the Con1 strain (genotype 1b). Based on predictive computer analysis and data from the literature, we extended the canonical TM regions in order to guarantee the exchange of only the soluble part of the E1 and E2 proteins, thus maintaining potential membranotropic domains. The completely secreted forms of E1 and E2 are truncated in their C-terminus at position 311 and 661, respectively, (or position 668 in strain 1a) [42] although it has been claimed that E1 has an internal TM that could allow the adoption of a polytopic form [43, 44]. In any case, there is a general consensus that the position of the TM region starts at position 352 and 715 for E1 and E2, respectively [45]. As a consequence, there is a gap of 40–50 residues that is responsible for the partial or complete retention of the two proteins into the ER even if it is not inserted in the membrane. According to this data, we swapped the unequivocally defined ectodomain portions (192–330 and 384–683, resp., for E1 and E2) and maintained the TM and the pre-TM regions of JFH1 (i.e., 312–330 and 669–683 for E1 and E2, resp.). For these JFH1 regions, the structure prediction of the N-terminus part is compatible with an amphipathic *α*-helix lying almost parallel with the membrane, establishing hydrophobic interactions with the lipid bilayer and exposing upward facing hydrophilic residues. Although this prediction has not been experimentally confirmed yet, the overall architecture of the TM and pre-TM regions of E1E2 would be very similar to that described for the ME complex of the Dengue Virus [46].

Analysis of the described chimeras, named JFH/Con1E1E2 and JFH1/Con1E2, was performed in parallel with the defective genome JFH1ΔE1E2 (without the E1 and E2 coding sequence) and with JFH1 wt. As expected, the E1 and E2 envelope proteins are not involved in the HCV genome replication process, since both chimeric constructs, as well as the defective genome, are able to efficiently replicate in S6.1 hepatoma cells. This observation is in agreement with the common role assigned to E1 and E2 proteins. Indeed, their predominant function as components of the viral spikes make them crucial for HCV entry into target cell, but dispensable for RNA replication [47, 48]. Conversely, it is quite surprising that none of the chimeric HCV genomes allow the production of infectious viral particles and behave like the ΔE1E2 defective RNA in this respect. Although it was already observed that deletion of the two glycoproteins from the viral genome impairs core secretion, which is an index for viral particle production [5], here, we extend this data and determined that even the heterodimer formation is not sufficient for virus production. In the attempt to explain this issue, we focused our attention on the JFH1/Con1E2 chimeric construct, in which only the E2 ectodomain portion is swapped. It has recently been demonstrated that E1 can associate with core protein through a cytoplasmic loop of five amino acids that show a high variability between genotypes 2a and 1b [22]. By using the JFH1/Con1E2 construct, we could exclude the possibility that the Con1-derived E1 protein is simply unable to interact with the capsid protein. Moreover, this chimera allowed us to obtain more useful information on the role of the E2 glycoprotein in HCV morphogenesis. To rule out a nonfunctional interaction between GT2a E1 and GT1b E2 glycoproteins, we first analyzed the folding process of the E1E2 heterodimers. In our analysis, the chimeric E1E2 complex reaches the same maturation as the wt heterodimers, even though the pathway is slightly delayed in protein precursor processing, oxidation kinetics, and E1E2 association. It is unlikely that a slower maturation process could lead to a complete loss of functionality, and different kinetics have already been described as genotype-associated in studies with recombinant proteins [7]. Therefore, the presence of chimeric heterodimers prompted us to evaluate subsequent steps in the viral morphogenesis, in particular the assembly process. 

Confocal microscopy analysis firstly suggested that chimeric particle assembly is in some way affected by the E2 ectodomain exchange. In fact, although core, E1E2 and NS5a proteins mostly display the same subcellular localization pattern in S6.1/JFH1 and S6.1/Con1E2 cells, we noted a peripheral accumulation of lipid droplets (LDs) coated by the capsid protein in almost all cells transfected with the chimeric genome. LDs were recently discovered as the site of HCV assembly [32], and their localization is usually found as perinuclear. The observed accumulation of core-coated LDs at the periphery of the S6.1/Con1E2 cells appears to be a consequence of a defective assembly process and could indicate a block in the secretion of competent, although immature, particles. In fact, it has been described that the production of extracellular infectious virus is preceded by the accumulation of intracellular immature infectious precursors that reach their final configuration along their egress [36, 37]. These particles are revealed as infectious species following cell rupture by freeze-thaw treatments. As we did not detect immature infectious precursor within S6.1/Con1E2 cells, we can conclude that the role of E2 in particle assembly is relevant early in morphogenesis. An additional observation supporting the accumulation of nonfunctional structures is the copresence of viral structural and nonstructural proteins in dense pointed structures, noted as dots, in the chimeric RNA-transfected cells. Presumably, these dots represent sites in which the viral proteins are stored instead of being released as mature particles. It is worth mentioning that in the replicon system the co-localization of viral structural and nonstructural proteins in dot-like structures was commonly detected [33]. In that context, it was suggested that dots represent HCV prebudding areas where all the viral proteins and RNA accumulate on ER-derived membranes. So far, our results argue against this conclusion, since we did not detect any similar co-localization of E1E2, core, and NS5a proteins into virus-producing cells. Since the replicon system, as well as S6.1/Con1E2 cells, did not allow the production of infectious viral particles, we hypothesize that dots are the result of an inefficient assembly process rather then a prebudding area. 

The observation that in JFH1/Con1E2-cells infectious particles were secreted following transcomplementation with the genotype 2a E2 protein further indicates that E2 acts in a strain-specific context. For HCV, it is already known that the entire structural region, from the core to the N-terminus of NS2, can be transcomplemented [41] whilst all viral proteins involved in RNA replication, with the exception of NS5A, cannot be provided *in trans* [34]. Providing the genotype 2a E2 (i.e., from the same isolate as the remaining structural region), we were able to rescue the virus production abolished by the E2 ectodomain exchange. The virus titers reached with the JFH1/Con1E2 genome complemented *in trans* are lower than that obtained with the JFH1 wt RNA, but they are identical to those obtained following *trans* complementation of ΔE1E2 defective genome. This observation strongly indicates that the infectious particles are most likely decorated with the wild type E1E2 heterodimers provided by the packaging cell. Unfortunately, the lacking of genotype-specific anti-E2 antibodies did not allow us to distinguish between wild type and chimeric HCV particles. 

The swapping of the E2 ectodomain could abolish virus production in two possible ways. The introduction of a different E2 domain, although taken from an infectious strain, could alter the fine balance of events that precedes the assembly of viral particles by impairing the formation of structures of a higher order than the heterodimer thus completely eliminating their infectious ability. Indeed, although we have demonstrated that chimeric E1E2 can associate, the sedimentation analysis of E2 complexes indicated an abundance of heterogeneous species in the chimeric case. Thus, it is possible that although E1E2 heterodimers can achieve a certain degree of native conformation, they can not progress to viral incorporation due to the lack of a fully competent state. Another possibility is that E2 ectodomain influences the cell culture-produced HCV by interacting with one or more of the other viral proteins (e.g., core, p7 or NS2) during the viral assembly process. This hypothesis is in agreement with several lines of evidence. Firstly, all the chimeric JFH1-based viruses successfully obtained contain the entire genotype 2a structural region, from the core to the NS2 protein [3, 4, 7], overcoming the potential incompatibility among structural proteins (including p7 and NS2) derived from different genotypes. Furthermore, it is worth mentioning that a genotypic incompatibility has already been observed for other HCV proteins, namely, p7 and NS2 [3, 4, 7, 49]. In particular, it was demonstrated that both p7 and NS2 proteins are essential for infectious particle assembly and release, and that their function most likely implies genotypic-specific interactions with other structural proteins. Pietschmann and coworkers also suggested that an interaction between E2 and NS2 might be important in HCV morphogenesis [7]. Our results are in line with this observation and add the information that such interaction requires a precise region of the E2 protein, namely, the ectodomain portion. Alternatively, the genotype-specific recognition of the E2 ectodomain could involve core or p7 proteins. We do not favor the latter possibility as the small polypeptide p7 is embedded in the ER membrane, and thus its interaction with the soluble portion of E2 is unlikely. For the same reason, the interaction with core protein is highly underprivileged since the E2 ectodomain is oriented towards the ER-lumen, whilst the core protein resides in the cytoplasm. Conversely, the relative positions of the E2 ectodomain and NS2 in the ER membrane argue for their interaction. Although the precise topology of NS2 is not yet defined, one possibility is that its soluble portion is ER-lumen exposed [50] and thus available for the interaction with the envelope protein.

In conclusion, we demonstrated that the soluble region of the HCV-E2 glycoprotein is essential for the production of infectious particles in the HCVcc system and suggest that it acts in concert with the other viral structural proteins at early stage of viral assembly in a genotypic-specific way.

## Supplementary Material

Figure S1 — Cartoon of HCV envelope glycoproteins as lying on the ER membrane. The ectodomain portion of both E1 and E2 is exposed on the lumen of the endoplasmic reticulum. The proposed TM regions are indicated as squared aminoacid position, while those reported in literature as not-squared aminoacid position. Pre-TM are illustrated as *α*-helix that precede the domains completely spanning the ER-membrane. Alignment of JFH1 and Con1 envelope E1 and E2 proteins is reported below. Aminoacids are numbered from the beginning of E1 to the end of E2. Underlined aminoacids represent the transmembrane regions. (∗) refers to identical aminoacids, (*+*) refers to chemically similar aminoacids.
Figure S2 — PCR-based strategy of Con1E1E2 and Con1E2 chimeric constructs Con1 sequence is represented as white boxes while JFH1 sequence in grey; the transmembrane region (TM) of both genotypes is represented as speckled box.
(A) To swap the E1 protein ectodomain, full-length replicon I389neo/core-30/5.1 was used as template. A forward primer consisting of a 5'-tail of 15 nt homologous to the end of JFH1 core protein and the 3'-portion complementary to the beginning of E1 Con1-derived (f1) was used in combination with four different reverse primers (from r2 to r5). These primers were spanning the extremity of the E1 ectodomain region of Con1 and the entire E1 TM of JFH1 in order to progressively swap the E1 ectodomain from genotype 2a to 1b. Similarly for E2 protein, a reverse primer consisting of 20nt complementary to the end of E2 ectodomain derived from Con1 strain plus a tail homologous to the beginning of the E2 TM region from JFH1 sequence (r1) was used in combination with four forward primers complementary to the E1 TM region JFH1-dedrived (from f2 to f5). In this way from PCR1 was obtained a chimeric fragment consisting on both E1 and E2 proteins of GT1b with the exception of the TM portion of E1 and two tails at the 5' and 3'-end that derive from JFH1 sequence. PCR2 and PCR3 were performed to amplify the 5'NTR-core and the E2 TM-NS2 segments of JFH1 genome using pUC.JFH1 plasmid as template. For PCR2 were used a T7 forward primer (fT7) and a reverse primer consisting of the 3'-end complementary to the end of JFH1 core coding region and the 5'-end homologous to the beginning of E1 Con1-derived (r6). Therefore it was obtained a fragment comprising the T7 promoter sequence (to permit generation of in *vitro* transcript), the JFH1 5'NCR and the entire core sequence plus a 15nt tail complementary to the beginning of E1 protein Con1-derived. In PCR3 the forward primer in part anneals to the start of GT2a-E2 TM region and the first 15nt are homologous to the end of E2 ectodomain GT1b-derived (f6). As reverse primer it was used an oligo complementary to NS2 protein from JFH1 sequence (rNS2). From this PCR was thus obtained a fragment consisting of E2 TM region, p7 sequence, and part of NS2 coding region JFH1-derived. At 5'-end of such fragment is also present a tail of 15nt homologous to the end of the E2 ectodomain derived from Con1 sequence. Finally, the products obtained from PCR1, 2 and 3 were fused in a single DNA molecule (shown as PCR4) using as forward primer an oligo complementary to T7 promoter (fT7bis) and as reverse an oligo complementary to the NS2 region JFH1-derived (rNS2bis). The chimeric JFH1/Con1E1E2 fragment was then cloned into pUC.JFH1 plasmid between the AgeI and NotI restriction sites replacing the same T7-NS2 region.
(B) To generate plasmid pUC.JFH/Con1E2, the pUC.JFH/Con1E1E2 was digested with EcoRI and BsiwI restriction obtaining a fragment spanning the 5'NCR-E1 TM region. The product was then replaced with the corresponding derived from JFH1 full length genome. In that way, in pUC.JFH/Con1E2 only the E2 ectodomain region is swapped from JFH1 to Con1 sequence.
Forward primers:
f1: 5' TCACCGTTCCGGTCTCTGCTTATGAAGTGCGCAAC 3'
f2: 5' TCATCCTTCTGCTGGCCGCTGGCGTTGACGGGGGA 3'
f3: 5' AGCGTGGGCGAAGGTCATTGTCATCCTTCTGCTGG 3'
f4: 5' GCCTACTTCTCTATGCAGGGAGCGTGGGCGAAGGT 3'
f5: 5' GGGGCGTCATGTTCGGCTTGGCCTACTTCTCTATG 3'
fT7: : 5' CCATAGTGGTCTGCGG 3'
f6: 5' CCCTGTTCCTTCACCACCCTACCCGCTTTGTCAACT 3'
fT7bis: 5' TTCCCAAACGCGTTAATAC 3'
Reverse primers:
r1: 5' AGACCAGTTGACAAAGCGGGTAGGGTGGTGAAGGA 3'
r2: 5' TACGCCAGGATCATGGTGGC TGTAGGTGACCAGTT 3'
r3: 5'CTCGGGGACGCGCATCACGTACGCCAGGATCATGG 3'
r4: 5' CTAACGATGTCTATGATGACCTCGGGGACGCGCAT 3'
r5: 5' ATGACGCCCCAGTGAGCCCCGCTAACGATGTCTATG 3'
r6: 5' GATACGTTGCGCACTTCATAAGCAGAGACCGGAAC 3'
rNS2: : 5' TGACGGCCCACGCGATGC 3'
rNS2bis: 5' TGTCAAACACCACACCCG 3'

## List of Abbreviations

ORF: Open reading frame
GT: Genotype
JFH: Japanese fulminant hepatitis
ER: Endoplasmic reticulum
DTT: Dithiothreitol
PNS: Post nuclear supernatant.

## References

[1] Lindenbach BD, Rice CM (2003). Molecular biology of flaviviruses. *Advances in Virus Research*.

[2] Bartenschlager R, Lohmann V (2000). Replication of the hepatitis C virus. *Bailliere’s Best Practice and Research in Clinical Gastroenterology*.

[3] Steinmann E, Penin F, Kallis S, Patel AH, Bartenschlager R, Pietschmann T (2007). Hepatitis C virus p7 protein is crucial for assembly and release of infectious virions.. *PLoS Pathogens*.

[4] Jones CT, Murray CL, Eastman DK, Tassello J, Rice CM (2007). Hepatitis C virus p7 and NS2 proteins are essential for production of infectious virus. *Journal of Virology*.

[5] Wakita T, Pietschmann T, Kato T (2005). Production of infectious hepatitis C virus in tissue culture from a cloned viral genome. *Nature Medicine*.

[6] Gottwein JM, Scheel TKH, Jensen TB (2009). Development and characterization of hepatitis C virus genotype 1-7 cell culture systems: role of CD81 and scavenger receptor class B type I and effect of antiviral drugs. *Hepatology*.

[7] Pietschmann T, Kaul A, Koutsoudakis G (2006). Construction and characterization of infectious intragenotypic and intergenotypic hepatitis C virus chimeras. *Proceedings of the National Academy of Sciences of the United States of America*.

[8] Deleersnyder V, Pillez A, Wychowski C (1997). Formation of native hepatitis C virus glycoprotein complexes. *Journal of Virology*.

[9] Cocquerel L, Wychowski C, Minner F, Penin F, Dubuisson J (2000). Charged residues in the transmembrane domains of hepatitis C virus glycoproteins play a major role in the processing, subcellular localization, and assembly of these envelope proteins. *Journal of Virology*.

[10] Dubuisson J (2007). Hepatitis C virus proteins. *World Journal of Gastroenterology*.

[11] Goffard A, Callens N, Bartosch B (2005). Role of N-linked glycans in the functions of hepatitis C virus envelope glycoproteins. *Journal of Virology*.

[12] Zhu Q, Oei Y, Mendel DB (2006). Novel robust hepatitis C virus mouse efficacy model. *Antimicrobial Agents and Chemotherapy*.

[13] Grakoui A, Hanson HL, Rice CM (2001). Bad time for Bonzo? Experimental models of hepatitis C virus infection, replication, and pathogenesis. *Hepatology*.

[14] Pietschmann T, Lohmann V, Kaul A (2002). Persistent and transient replication of full-length hepatitis C virus genomes in cell culture. *Journal of Virology*.

[15] Huang L (2005). Hepatitis C virus nonstructural protein 5A (NS5A) is an RNA-binding protein. *Journal of Biological Chemistry*.

[16] Zhong J, Gastaminza P, Cheng G (2005). Robust hepatitis C virus infection in vitro. *Proceedings of the National Academy of Sciences of the United States of America*.

[17] Braakman I, Hoover-Litty H, Wagner KR, Helenius A (1991). Folding of influenza hemagglutinin in the endoplasmic reticulum. *Journal of Cell Biology*.

[18] Hadlock KG, Lanford RE, Perkins S (2000). Human monoclonal antibodies that inhibit binding of hepatitis C virus E2 protein to CD81 and recognize conserved conformational epitopes. *Journal of Virology*.

[19] Tarentino AL, Plummer TH (1994). Enzymatic deglycosylation of asparagine-linked glycans: purification, properties, and specificity of oligosaccharide-cleaving enzymes from Flavobacterium meningosepticum. *Methods in Enzymology*.

[20] Hope RG, McLauchlan J (2000). Sequence motifs required for lipid droplet association and protein stability are unique to the hepatitis C virus core protein. *Journal of General Virology*.

[21] Rouillé Y, Helle F, Delgrange D (2006). Subcellular localization of hepatitis C virus structural proteins in a cell culture system that efficiently replicates the virus. *Journal of Virology*.

[22] Nakai K, Okamoto T, Kimura-Someya T (2006). Oligomerization of hepatitis C virus core protein is crucial for interaction with the cytoplasmic domain of E1 envelope protein. *Journal of Virology*.

[23] Ciczora Y, Callens N, Penin F, Pécheur E-I, Dubuisson J (2007). Transmembrane domains of hepatitis C virus envelope glycoproteins: residues involved in E1E2 heterodimerization and involvement of these domains in virus entry. *Journal of Virology*.

[24] Goffard A, Dubuisson J (2003). Glycosylation of hepatitis C virus envelope proteins. *Biochimie*.

[25] Brazzoli M, Helenius A, Foung SKH, Houghton M, Abrignani S, Merola M (2005). Folding and dimerization of hepatitis C virus E1 and E2 glycoproteins in stably transfected CHO cells. *Virology*.

[26] Choukhi A, Pillez A, Drobecq H, Sergheraert C, Wychowski C, Dubuisson J (1999). Characterization of aggregates of hepatitis C virus glycoproteins. *Journal of General Virology*.

[27] Braakman I, Helenius J, Helenius A (1992). Manipulating disulfide bond formation and protein folding in the endoplasmic reticulum. *EMBO Journal*.

[28] Op De Beeck A, Cocquerel L, Dubuisson J (2001). Biogenesis of hepatitis C virus envelope glycoproteins. *Journal of General Virology*.

[29] Shavinskaya A, Boulant S, Penin F, McLauchlan J, Bartenschlager R (2007). The lipid droplet binding domain of hepatitis C virus core protein is a major determinant for efficient virus assembly. *Journal of Biological Chemistry*.

[30] El-Hage N, Luo G (2003). Replication of hepatitis C virus RNA occurs in a membrane-bound replication complex containing nonstructural viral proteins and RNA. *Journal of General Virology*.

[31] Shi ST, Lee K-J, Aizaki H, Hwang SB, Lai MMC (2003). Hepatitis C virus RNA replication occurs on a detergent-resistant membrane that cofractionates with caveolin-2. *Journal of Virology*.

[32] Miyanari Y, Atsuzawa K, Usuda N (2007). The lipid droplet is an important organelle for hepatitis C virus production. *Nature Cell Biology*.

[33] Brazzoli M, Crotta S, Bianchi A (2007). Intracellular accumulation of hepatitis C virus proteins in a human hepatoma cell line. *Journal of Hepatology*.

[34] Appel N, Zayas M, Miller S (2008). Essential role of domain III of nonstructural protein 5A for hepatitis C virus infectious particle assembly. *PLoS Pathogens*.

[35] Shi ST, Polyak SJ, Tu H, Taylor DR, Gretch DR, Lai MMC (2002). Hepatitis C virus NS5A colocalizes with the core protein on lipid droplets and interacts with apolipoproteins. *Virology*.

[36] Gastaminza P, Cheng G, Wieland S, Zhong J, Liao W, Chisari FV (2008). Cellular determinants of hepatitis c virus assembly, maturation, degradation, and secretion. *Journal of Virology*.

[37] Gastaminza P, Kapadia SB, Chisari FV (2006). Differential biophysical properties of infectious intracellular and secreted hepatitis C virus particles. *Journal of Virology*.

[38] Merola M, Brazzoli M, Cocchiarella F (2001). Folding of hepatitis C virus E1 glycoprotein in a cell-free system. *Journal of Virology*.

[39] Rosa D, Campagnoli S, Moretto C (1996). A quantitative test to estimate neutralizing antibodies to the hepatitis C virus: cytofluorimetric assessment of envelope glycoprotein 2 binding to target cells. *Proceedings of the National Academy of Sciences of the United States of America*.

[40] Liljeström P, Garoff H (1991). A new generation of animal cell expression vectors based on the semliki forest virus replicon. *Nature Biotechnology*.

[41] Steinmann E, Brohm C, Kallis S, Bartenschlager R, Pietschmann T (2008). Efficient trans-encapsidation of hepatitis C virus RNAs into infectious virus-like particles. *Journal of Virology*.

[42] Michalak J-P, Wychowski C, Choukhi A (1997). Characterization of truncated forms of hepatitis C virus glycoproteins. *Journal of General Virology*.

[43] Matsuura Y, Harada T, Makimura M (1994). Characterization of HCV structural proteins expressed in various animal cells. *Intervirology*.

[44] Migliaccio CT, Follis KE, Matsuura Y, Nunberg JH (2004). Evidence for a polytopic form of the E1 envelope glycoprotein of hepatitis C virus. *Virus Research*.

[45] Lavie M, Goffard A, Dubuisson J (2007). Assembly of a functional HCV glycoprotein heterodimer. *Current Issues in Molecular Biology*.

[46] Zhang W, Chipman PR, Corver J (2003). Visualization of membrane protein domains by cryo-electron microscopy of dengue virus. *Nature Structural Biology*.

[47] Bartosch B, Bukh J, Meunier J-C (2003). In vitro assay for neutralizing antibody to hepatitis C virus: evidence for broadly conserved neutralization epitopes. *Proceedings of the National Academy of Sciences of the United States of America*.

[48] Nielsen SU, Bassendine MF, Burt AD, Bevitt DJ, Toms GL (2004). Characterization of the genome and structural proteins of hepatitis C virus resolved from infected human liver. *Journal of General Virology*.

[49] Haqshenas G, Dong X, Ewart G, Bowden S, Gowans EJ (2007). A 2a/1b full-length p7 inter-genotypic chimeric genome of hepatitis C virus is infectious in vitro. *Virology*.

[50] Yamaga AK, Ou J-H (2002). Membrane topology of the hepatitis C virus NS2 protein. *Journal of Biological Chemistry*.

