# Supplementary material for: Hepatitis C Virus E2 Protein Ectodomain Is Essential for Assembly of Infectious Virions
Source: Int J Hepatol. 2010 Oct 12;2011:968161. doi: 10.4061/2011/968161 (PMC3172978; doi:10.4061/2011/968161)
Supplement: Supplementary file 1 — Figure S1 — Cartoon of HCV envelope glycoproteins as lying on the ER membrane. The ectodomain portion of both E1 and E2 is exposed on the lumen of the endoplasmic reticulum. The proposed TM regions are indicated as squared aminoacid position, while those reported in literature as not-squared aminoacid position. Pre-TM are illustrated as α-helix that precede the domains completely spanning the ER-membrane. Alignment of JFH1 and Con1 envelope E1 and E2 proteins is reported below. Aminoacids are numbered from the beginning of E1 to the end of E2. Underlined aminoacids represent the transmembrane regions. (∗) refers to identical aminoacids, (+) refers to chemically similar aminoacids. Figure S2 — PCR-based strategy of Con1E1E2 and Con1E2 chimeric constructs Con1 sequence is represented as white boxes while JFH1 sequence in grey; the transmembrane region (TM) of both genotypes is represented as speckled box. (A) To swap the E1 protein ectodomain, full-length replicon I389neo/core-30/5.1 was used as template. A forward primer consisting of a 5'-tail of 15 nt homologous to the end of JFH1 core protein and the 3'-portion complementary to the beginning of E1 Con1-derived (f1) was used in combination with four different reverse primers (from r2 to r5). These primers were spanning the extremity of the E1 ectodomain region of Con1 and the entire E1 TM of JFH1 in order to progressively swap the E1 ectodomain from genotype 2a to 1b. Similarly for E2 protein, a reverse primer consisting of 20nt complementary to the end of E2 ectodomain derived from Con1 strain plus a tail homologous to the beginning of the E2 TM region from JFH1 sequence (r1) was used in combination with four forward primers complementary to the E1 TM region JFH1-dedrived (from f2 to f5). In this way from PCR1 was obtained a chimeric fragment consisting on both E1 and E2 proteins of GT1b with the exception of the TM portion of E1 and two tails at the 5' and 3'-end that derive from JFH1 sequence. PCR2 and PCR3 were [file 968161.f1.pdf]

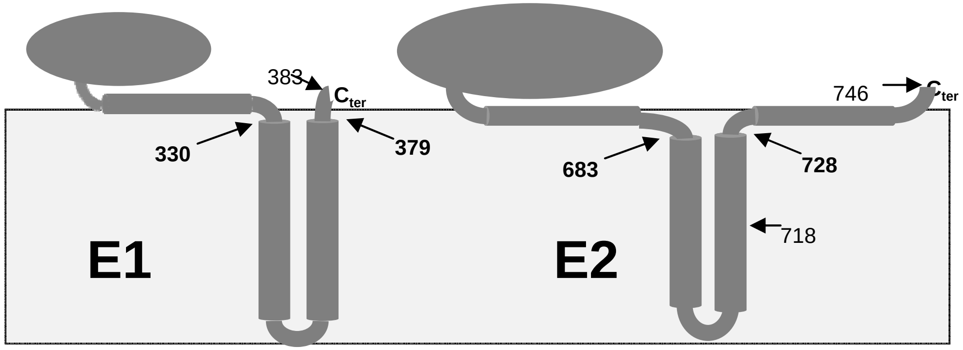


```
JFH1  192    AQVKNTSSSYMVTNDCSNDSITWQLEAAVLHVPGCVPCERVGNTSRCWVPVSPNMAVRQPG  252
              +*+* *   * ******* ** ++     ++* ****** *   *+***** ++* +* *
Con1  192    YEVRNVSGVYHVTNDCSNASIVYEAADMIMHTPGCVPCVRENNSSRCWVALTPTLAARNAS  252

JFH1  253    ALTQGLRTHIDMVVMSATFCSALYVGDLCGGVMLAAQVFIVSPQYHWFVQECNCSIYPGT  312
               *  +* *+*++* +*  ***+******* * * **+*  **+ *  **+********
Con1  253    VPTTTIRRHVDLLVGAAALCSAMYVGDLCGSVFLVAQLFTFSPRRHETVQDCNCSIYPGH  312

JFH1  313    ITGHRMAWDMMMNWSPTATMILAYVMRVPEVIIDIVSGAHWGVMFGLAYFSMQGAWAKVI  372
             +***************** ++++ ++*+*+ ++*+*+******+ ****+** * ****+
Con1  313    VTGHRMAWDMMMNWSPTAALVVSQLLRIPQAVVDMVAGAHWGVLAGLAYYSMVGNWAKVL  372

JFH1  373    VILLLAAGVDA  383
             +++** ****
Con1  373    IVMLLFAGVDG  383
```

```
JFH1   384  GTTTVGGAVARSTNVIAGVFSHGPQQNIQLINTNGSWHINRTALNCNDSLNTGFLAALFY  443
            **   ** +*++*  *  +** *  * ***+******************************
Con1..384  GTYVTGGTMAKNTLGITSLFSPGSSQKIQLVNTNGSWHINRTALNCNDSLNTGFLAALFY  443

JFH1   444  TNRFNSSGCPGRLSACRNIEAFRIGWGTLQYEDNVTNPEDMRPYCWHYPPKPCGVVPARS  503
             ++******* *+++*  *+**  *** + *  * ++  * ******* *+***+***
Con1   444  VHKFNSSGCPERMASCSPIDAFAQGWGPITY--NESHSSDQRPYCWHYAPRPCGIVPAAQ  501

JFH1   504  VCGPVYCFTPSPVVVGTTDRRGVPTYTWGENETDVFLLNSTRPPQGSWFGCTWMNSTGFT  563
            ******************** *****+******** ***+******+*************
Con1   502  VCGPVYCFTPSPVVVGTTDRFGVPTYSWGENETDVLLLNNTRPPQGNWFGCTWMNSTGFT  561

JFH1   564  KTCGAPPCRTRADFNASTDLLCPTDCFRKHPDATYIKCGSGPWLTPKCLVHYPYRLWHYP  623
            **** ***      * +  * *********+*** **********+*************
Con1   562  KTCGGPPCNIGGIGNKT--LTCPTDCFRKHPEATYTKCGSGPWLTPRCLVHYPYRLWHYP  619

JFH1   624  CTVNFTIFKIRMYVGGVEHRLTAACNFTRGDRCDLEDRDRSQLSPLLHSTTEWAILPCTY  683
            *********+*********** ****+***+**+*******+***** ***** +***++
Con1   620  CTVNFTIFKVRMYVGGVEHRLEAACNWTRGERCNLEDRDRSELSPLLLSTTEWQVLPCSF  679

JFH1   684  SDLPALSTGLLHLHQNIVDVQYMYGLSPAITKYVVRWEWVVLLFLLLADARVCACLWMLI  743
             +********+*****+*****+**+  *+  + ++**+*+*****************++
Con1   680  TTLPALSTGLIHLHQNVVDVQYLYGIGSAVVSFAIKWEYVLLLFLLLADARVCACLWMML  739

JFH1   744  LLGQAEA  750
            *+ ****
Con1   740  LIAQAEA  746
```
