# Supplementary figures and images for: Hepatitis C Virus E2 Protein Ectodomain Is Essential for Assembly of Infectious Virions
Source: Int J Hepatol. 2010 Oct 12;2011:968161. doi: 10.4061/2011/968161 (PMC3172978; doi:10.4061/2011/968161)

A.

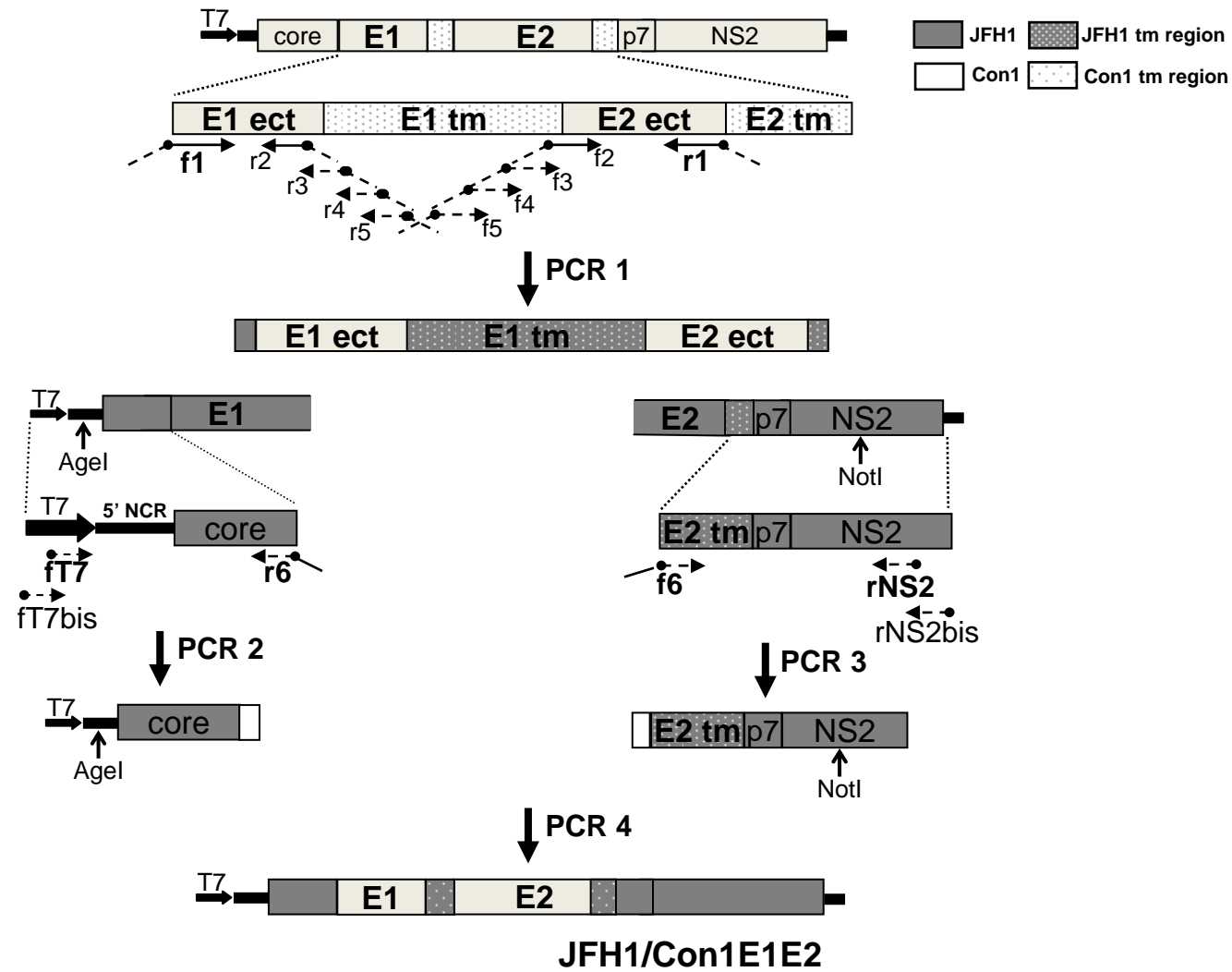

**B.**

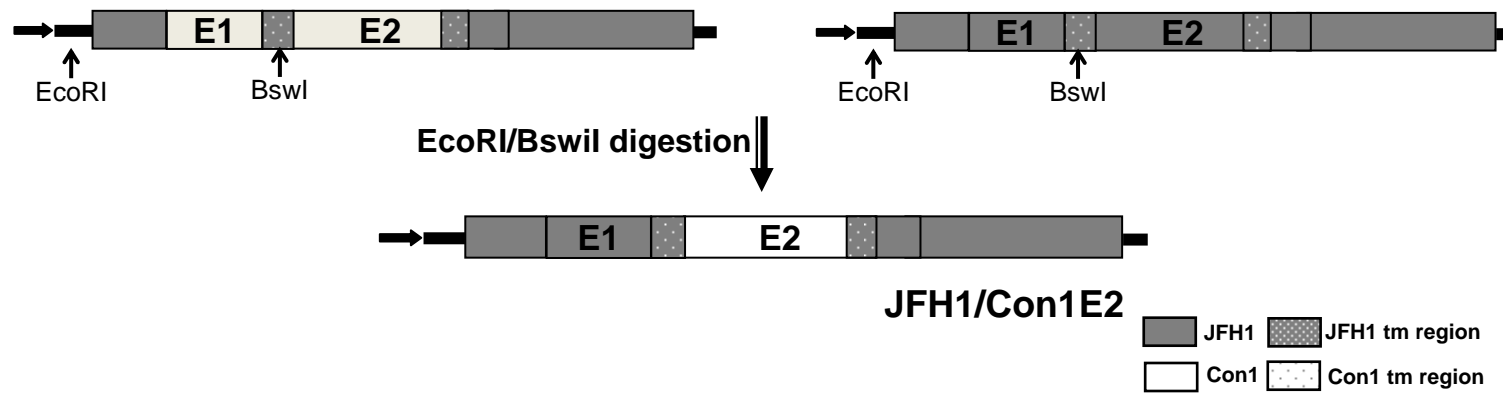

Supplement: Supplementary file 2 [file 968161.f2.pdf]
